# To What Extent Do Financial Strain and Labour Force Status Explain Social Class Inequalities in Self-Rated Health? Analysis of 20 Countries in the European Social Survey

**DOI:** 10.1371/journal.pone.0110362

**Published:** 2014-10-14

**Authors:** Richard J. Shaw, Michaela Benzeval, Frank Popham

**Affiliations:** 1 Medical Research Council/Chief Scientist Office Social and Public Health Sciences Unit, University of Glasgow, Glasgow, United Kingdom; 2 Institute for Social and Economic Research, University of Essex, Colchester, United Kingdom; Brighton and Sussex Medical School, United Kingdom

## Abstract

**Introduction:**

Nordic countries do not have the smallest health inequalities despite egalitarian social policies. A possible explanation for this is that drivers of class differences in health such as financial strain and labour force status remain socially patterned in Nordic countries.

**Methods:**

Our analyses used data for working age (25–59) men (n = 48,249) and women (n = 52,654) for 20 countries from five rounds (2002–2010) of the European Social Survey. The outcome was self-rated health in 5 categories. Stratified by gender we used fixed effects linear regression models and marginal standardisation to instigate how countries varied in the degree to which class inequalities were attenuated by financial strain and labour force status.

**Results and Discussion:**

Before adjustment, Nordic countries had large inequalities in self-rated health relative to other European countries. For example the regression coefficient for the difference in health between working class and professional men living in Norway was 0.34 (95% CI 0.26 to 0.42), while the comparable figure for Spain was 0.15 (95% CI 0.08 to 0.22). Adjusting for financial strain and labour force status led to attenuation of health inequalities in all countries. However, unlike some countries such as Spain, where after adjustment the regression coefficient for working class men was only 0.02 (95% CI −0.05 to 0.10), health inequalities persisted after adjustment for Nordic countries. For Norway the adjusted coefficient was 0.17 (95% CI 0.10 to 0.25). Results for women and men were similar. However, in comparison to men, class inequalities tended to be stronger for women and more persistent after adjustment.

**Conclusions:**

Adjusting for financial security and labour force status attenuates a high proportion of health inequalities in some counties, particularly Southern European countries, but attenuation in Nordic countries was modest and did not improve their relative position.

## Introduction

In theory it was expected that the socio-economic gradient in health should be smallest in Nordic countries [1], [2] because their economic and social policies have aimed to make all class groups less reliant on market success for a high standard of financial welfare, while at the same time aiming for full employment [3]. As Esping Andersen argues “Perhaps the most salient characteristic of the social democratic regime is its fusion of welfare and work.” ([3]p.28). Thus social democratic Nordic welfare states, in comparison to other welfare regimes, should have smaller inequalities in key outcomes of social class relations, such as financial security and non-employment risk [4], which in turn ought to lead to smaller inequalities in health because financial security [5] and non-employment [6], [7] are important risk factors for poor health. However, unexpectedly this has tended to not be the case [8]–[11].

The reasons for the “failure” of the Nordic model to have the smallest health inequalities remain unexplained [1]. It has been postulated that one reason for this is that Nordic welfare states have not fully reduced inequalities in key mediators of the relationship between class and health [2]. There is also a debate around the extent to which the Nordic Welfare state model has changed in recent [12], [13]. It has been argued that whilst the core of the Nordic welfare state model remains their economies now require a more a dynamic, flexible and knowledge intensive labour force and this may have increased inequalities [12]. Hence Nordic welfare states may maintain significant levels of relative inequality even though overall levels of financial welfare and labour force participation may be high across all social class groups [3], [14]. In addition, Nordic countries operate on the principle of universalism which ensures that all class groups may potentially benefit [15], in contrast to other welfare models where the provision of services is more targeted to reduce inequalities.

In Bismarkian countries welfare programmes are relatively generous but linked to prior earnings thus maintaining pre-existing social patterns [6], [16]. Whilst in Southern European countries welfare provision is fragmented; generous in some areas but rudimentary in others, leaving people dependent on their families or the voluntary sector [6]. In Anglo Saxon countries welfare protection levels are modest and often attract strict entitlement criteria with recipients usually means-tested and potentially stigmatised [6]. Following the demise of the communist welfare states, Eastern European and Former Soviet countries [17] have adopted market-orientated polices associated with the Anglo-Saxon welfare state regimes [6]. In addition, people living in Eastern European and Former Soviet countries have the additional challenges of lower levels of wealth than people living in Western and Northern European countries [18], thus increasing the risk of financial insecurity.

Few studies have directly assessed financial security and labour force status as mediators of social class inequalities in health in a comparative setting. Aldabe et al [19] pooled data from 28 countries and found that financial problems, material deprivation, social exclusion and job reward were important mediators. However, this study had a relatively small sample size and did not investigate the importance of mediating factors varying across countries. Whilst Eikemo et al [20] conducted analyses that pooled 24 countries into 4 groups East, North, Central, and South and found that adjusting for education and income lead to a modest reduction in the effects of occupational social class, they did not investigate the impact for countries separately – an important consideration given that policies vary between countries within the same welfare regimes [21]. Further, measures of income, particularly measured at one point in time, may not adequately capture the living conditions that a person is experiencing [22]. In addition to income itself, it may be important to account for the demands placed on that income, which are likely to vary by the wider context in which a person lives. For example, a country's welfare state may mitigate deprivation even for those on lower incomes [22]. Measures of financial strain which capture adequacy of income, as used in this study, may be much more closely associated with welfare state type than are household income derived measures of poverty [14]. Financial strain has other advantages over reports of income, which can be difficult to measure accurately in social surveys. It is easy to record, explain and simple to interpret [23] and it has been shown to be more strongly associated with health than objective measures of household income. [24] Our aim then was to test the extent to which the ability of financial strain and labour force status to explain the relationship between social class and health differed between Nordic countries and other European countries using data from the European Social Survey (ESS).

## Methods

This study uses data for people aged 25 to 59 from the first 5 rounds (2002–2010) of the ESS which is a multi-national repeated cross-sectional survey [25]. We included participants that come from 20 countries that participated in at least 4 rounds of the ESS. These countries were Spain (ES), Greece (GR), Portugal (PT), UK (GB), Ireland (IE), Denmark, (DK), FI (Finland), Norway (NO), Sweden (SE), Belgium (BE), Switzerland (CH), Germany (DE), Netherlands (NL), Czechoslovakia (CZ), Hungary (HU), Poland (PL), Slovenia (SL), Slovakia (SK), Estonia (EE) and Ukraine (UA). We excluded France because the financial strain questions used by France in the first two rounds were not consistent with the other countries. The analytical sample contained 48,249 men and 52,654 women after excluding people from the sample who had missing data for self-rated health (0.1%), financial strain (0.7%), labour forces status (0.5%) and social class (4.2%). Targeted response rates for the ESS were 70%. The highest response (80%) was for Greece in round 1, whilst the lowest response rate (34%) was for Switzerland also in round 1. The data and extensive documentation are available from the ESS website (http://www.europeansocialsurvey.org/).

Self-rated health was assessed using a single question, translated into the appropriate language, which asked “How is your health in general?” with responses very good (5), good (4), fair (3), bad (2) or very bad (1). In this paper we treated self-rated health as a continuous variable which has been shown to be a reasonable assumption [26], [27]. We have also conducted analyses treating self-rated health as an ordinal variable and these provided very similar results.

Financial strain was classified using a single question which asked people how they felt about their household income with possible responses being 1 “Living comfortably on present income”, 2 “coping on present income”, 3 “finding it difficult on present income” and 4 “finding it very difficult on present income.”

Socio-economic class was assessed using the European Socio-economic Classification (ESeC) which is based on employment relations, reflects job and financial security and can be considered a continuation of the Erikson/Goldthorpe/Portocarero class scheme [28]. Participants who were not currently employed were asked about their previous employment. In order to have an ordinal scale and to avoid small numbers we used the established version with three categories – Salariat, Intermediate and working class.

Labour force status was assessed by asking participants which activity best describes his/her situation in the last 7 days. We used the categories in paid work, unemployed (including both those actively looking for a job and wanting a job but not actively looking), incapacitated (permanently sick or disabled), retired, looking after home (including caring for children and others) and other (including military/community service, education). In order to be consistent with the International Labour organisation classification [29] people who defined themselves as looking after their home and had also reported themselves as performing some paid work were classified as being employed. For Sweden we merged retired into the other category because very few people described themselves as being retired.

### Statistical analysis

To assess country variations in the degree to which financial strain and labour force status attenuated the relationship between social class and health measured at the level of the individual, we created four multilevel fixed effects linear regression models stratified by gender that account for the clustering of individual people within countries [30], [31]. In all models we included interaction terms between country and the independent variables of interest. Model 1, the base model, includes self-rated health as the dependent variable, social class (contrasting working and intermediate to salariat as reference category) as the main independent variable, and age and ESS round as covariates. In Model 2 financial strain is added to the base model. In Model 3 labour force status is added to the base model, whilst in Model 4 both financial strain and labour force status are included. In order to summarize how the associations between class and health vary between countries both before and after adjustment for financial strain and labour force status we used marginal standardisation [32], [33]. Additionally, we reran the base models using ordinal logistic regression and found no substantive differences in results. Analyses were conducted using Stata 13.1 and all presented analyses and figures are weighted using design weights to account for the sampling methodology used in each country.

## Results

Descriptive statistics are shown in Table 1 for men and Table 2 for women. Generally the distribution of self-reported health was similar for all countries within the same welfare regime. With the exception of Greece, the best health tended to be found in Anglo-Saxon and Nordic Countries. Bismarkian and Southern countries were in the middle whilst generally people in Eastern Europe and Former Soviet countries reported the lowest levels of very good health.

**Table 1:** Percentages for self-rated health, labour force status, financial strain and class and mean for age by country for men.

	ES	GR	PT	GB	IE	DK	FI	NO	SE	BE	CH	DE	NL	CZ	HU	PL	SI	SK	EE	UA
Number	2,682	2,346	1,974	2,858	2,641	2,194	2,812	2,872	2,630	2,506	2,597	4,030	2,639	2,327	2,021	2,472	1,753	1,747	1,624	1,524
	%	%	%	%	%	%	%	%	%	%	%	%	%	%	%	%	%	%	%	%
**Self-rated health**																				
Very Good	21	59	10	33	43	42	22	35	35	27	37	18	18	21	12	15	17	17	9	3
Good	55	31	58	44	44	38	49	46	48	56	51	49	63	49	46	50	48	51	43	39
Fair	20	9	26	18	11	15	25	14	14	14	10	26	16	24	31	28	29	27	41	50
Bad	3	1	5	5	1	3	3	4	2	3	2	7	3	5	9	7	5	5	6	7
Very Bad	0	1	1	1	0	1	1	1	1	0	0	1	0	1	2	1	1	1	1	1
**Labour force status**																				
Employed	86	85	84	83	79	86	84	89	90	85	91	84	88	88	77	77	80	84	83	78
Unemployed	9	9	8	6	12	4	6	3	4	5	3	8	3	5	9	9	6	9	8	8
Incapacitated	2	0	2	6	3	2	2	3	2	4	3	2	4	3	5	1	2	2	4	1
Retired	2	5	5	2	2	4	5	1	-	4	1	3	1	3	7	11	7	4	3	6
Looking after home	0	0	0	1	1	1	0	1	0	1	1	1	1	1	1	1	1	1	1	4
Other	1	1	1	2	3	4	4	3	4	1	2	3	3	1	2	1	4	1	2	2
**Financial Strain**																				
Living comfortably	35	10	9	38	35	69	21	57	60	40	54	30	52	15	6	7	42	14	10	2
Copping	47	39	61	45	46	26	66	35	33	42	36	53	39	54	52	57	45	52	60	28
Difficult	15	33	23	14	14	4	10	6	6	15	9	13	8	23	28	31	9	25	21	47
Very difficult	4	18	7	3	5	1	3	2	1	4	2	4	2	9	14	4	3	10	9	24
**Class**																				
Salariat	24	21	19	40	32	41	39	42	42	41	44	38	49	25	20	23	29	30	26	29
Intermediate	36	43	29	33	34	29	23	34	27	29	34	30	30	24	20	31	36	26	22	19
Working	41	36	52	27	35	30	38	24	31	30	22	32	21	51	60	46	35	44	53	52
**Age**																				
Mean	41	41	42	43	42	44	43	43	42	42	42	43	43	43	42	42	42	43	42	42
SD	10	10	10	10	10	10	10	10	10	10	10	9	10	10	10	10	10	10	10	10

**Table 2:** Percentages for self-rated health, labour force status, income strain and class and mean for age by country for women.

	ES	GR	PT	GB	IE	DK	FI	NO	SE	BE	CH	DE	NL	CZ	HU	PL	SI	SK	EE	UA
Number	2,534	2,543	2,770	3,347	3,215	2,213	2,752	2,560	2,557	2,475	2,885	4,048	3,255	2,313	2,312	2,521	1,861	2,071	2,055	2,367
	%	%	%	%	%	%	%	%	%	%	%	%	%	%	%	%	%	%	%	%
**Self-rated health**																				
Very Good	20	50	7	35	47	42	25	35	33	25	38	16	16	18	11	11	15	15	9	1
Good	49	34	49	42	40	39	51	44	45	54	49	50	59	49	42	48	45	49	44	25
Fair	25	13	36	18	10	15	21	15	17	17	11	27	21	26	36	33	34	30	40	59
Bad	5	2	7	4	2	4	2	4	4	3	2	6	4	6	9	7	6	5	6	14
Very Bad	1	1	1	1	1	1	0	1	1	1	1	1	1	1	2	1	1	1	1	2
**Econ activity**																				
Employed	71	65	69	72	60	80	77	82	83	72	75	71	71	71	65	62	71	70	77	61
Unemployed	9	11	13	3	5	5	6	3	5	7	2	7	2	6	6	8	6	7	6	7
Incapacitated	2	0	1	5	2	2	1	4	4	4	2	2	5	3	3	1	1	1	3	2
Retired	1	4	5	2	2	3	3	1	0	2	0	3	0	7	9	13	9	8	3	11
Household care	17	19	11	16	29	3	7	7	2	13	19	16	19	13	15	15	9	13	10	19
Other	1	1	2	3	3	6	5	4	7	2	2	3	3	1	2	1	4	1	1	1
**Financial Strain**																				
Living comfortably	33	9	8	38	35	66	24	53	55	38	50	28	48	11	6	6	37	10	10	2
Copping	46	37	53	44	45	28	63	38	35	40	39	54	39	52	48	56	48	48	54	19
Difficult	17	36	28	15	15	4	10	7	8	18	9	14	11	26	32	34	11	32	26	49
Very difficult	4	18	11	4	5	2	3	2	2	4	2	4	3	11	15	4	3	10	10	30
**Class**																				
Salariat	22	19	18	38	36	43	39	41	40	41	39	34	46	30	27	31	37	33	39	38
Intermediate	32	38	23	29	27	29	23	28	26	27	34	33	27	27	24	28	28	26	20	19
Working	47	43	59	34	37	28	38	32	34	33	28	33	27	43	50	42	36	41	42	43
**Age**																				
Mean	40	41	42	42	42	43	43	42	43	43	42	43	43	43	43	42	42	43	43	43
SD	9	9	10	10	10	10	10	10	10	10	10	9	10	10	10	10	10	10	10	10

**Figure 1:**
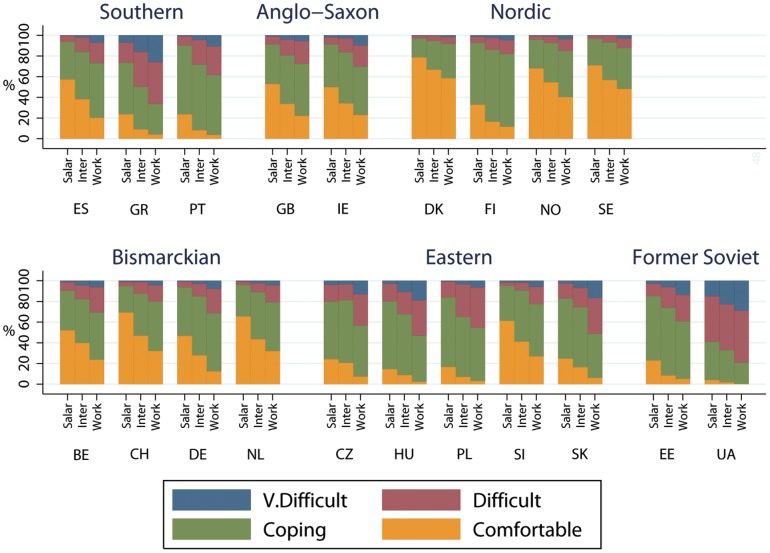
Financial strain by social class, country and welfare state for men.

**Figure 2:**
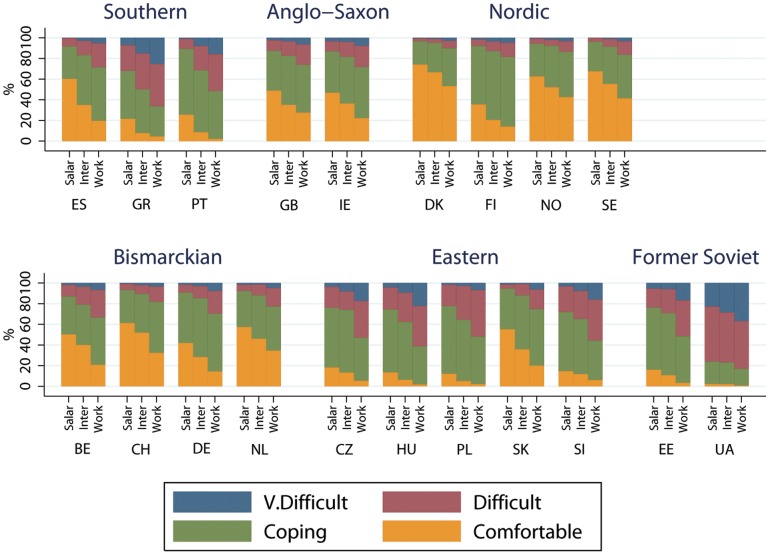
Financial strain by social class, country and welfare state for women.

Financial strain by class, country and welfare state is shown in Figure 1 for men and Figure 2 for women, with results by gender being very similar. In all countries there was a class gradient in financial strain, with the salariat reporting less strain than other classes. The highest percentage of financially comfortable people tended to be found in Nordic countries, followed by Bismarkian and Anglo-Saxon countries. Despite the low levels of financial strain in the latter countries, social class inequalities in strain were still apparent. In most Southern, Eastern and Former Soviet countries the highest proportion of people tended to be coping rather than comfortable, with higher proportions finding things difficult, and in Ukraine most people were finding things difficult or very difficult.

**Figure 3:**
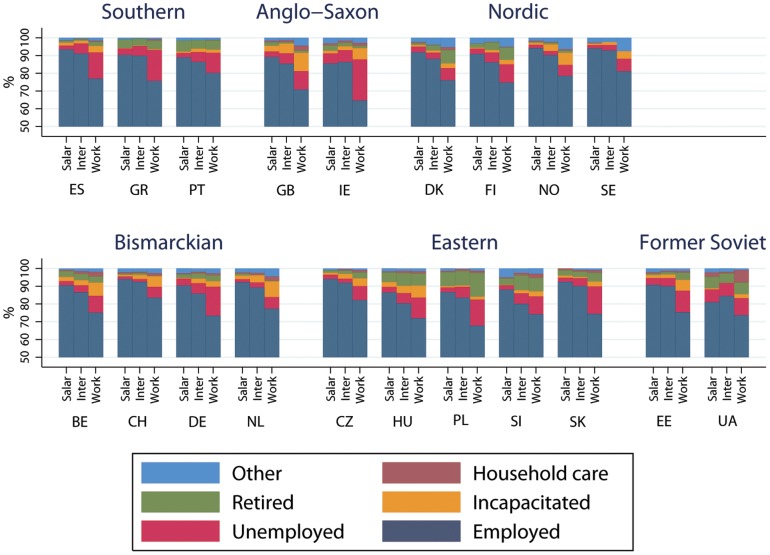
Labour force status by social class, country and welfare state for men.

**Figure 4:**
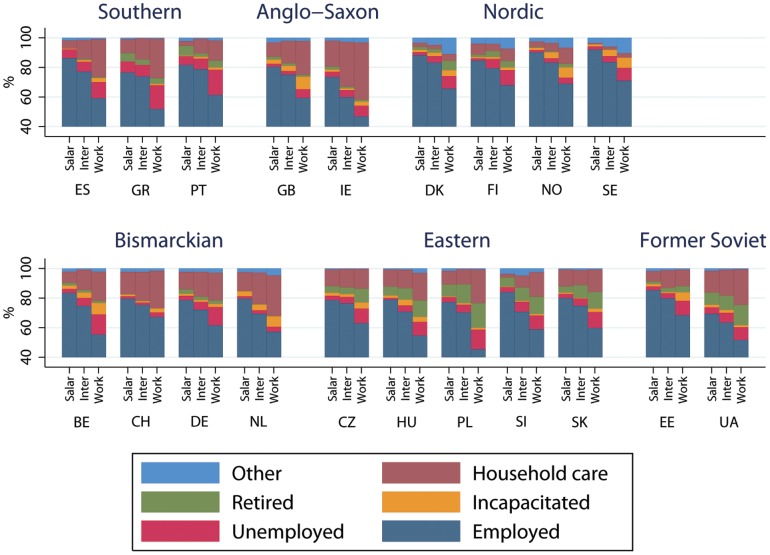
Labour force status by social class, country and welfare state for women.

Economic activity by class, country and welfare state is shown in Figure 3 for men and Figure 4 for women. The percentage of intermediate and salariat men employed are very similar. In nearly all Southern, Nordic and Bismarkian countries more than 90% of the Salariat are employed. In contrast for Anglo-Saxon, Eastern European and Former-Soviet countries employment rates for the salariat are typically less than 90%. Across Europe employment rates for working class people were substantially lower than the more advantaged classes and, with the exception of Portugal and Switzerland, in all countries fewer than 80% of working class men were employed. For men unemployment is the largest non-employed category. Women's employment rates are substantially lower than for men. Only in Nordic countries do both salariat and intermediate class women have employment rates greater than 80%. In addition, there is a gradient of decreasing employment across the three classes. For women, the largest non-employed category in most countries was looking after home and providing care. The exceptions to this are the Nordic countries where a comparatively high proportion of women were in the “other” category. In some Anglo-Saxon, Nordic and Bismarkian countries a relatively high proportion of working class people are out of work due to incapacity, whilst in Eastern Europe and Former Soviet countries a relatively high proportion report being retired.

**Figure 5:**
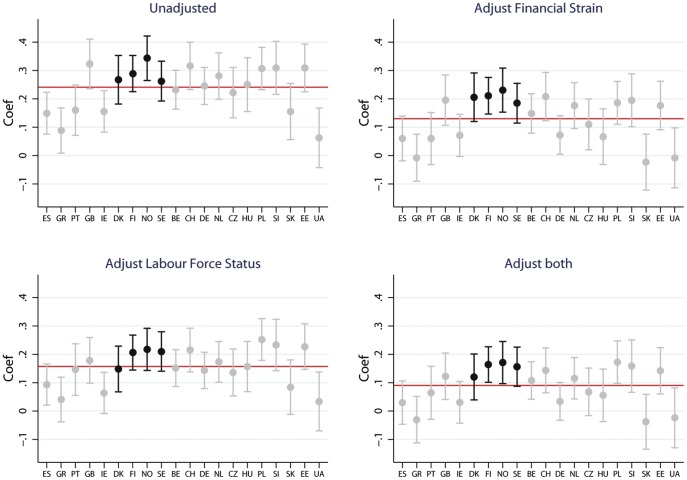
Self-rated health before and after adjustment, men. Figure 5 shows the predicted coefficients for self-rated health from marginal models for working class (relative to Salariat) for men for the entire sample (red line) and for 20 countries in the European Social Survey. Models include the base model, adjusting for financial strain, adjusting labour force status and adjusting for both labour force status and financial strain.

The coefficients for self-rated health of working class men relative to salariat for the entire sample and individual countries, from fixed effects regression models before and after adjusting for financial strain and labour force status, are shown in Figure 5. (The magnitude of the coefficient for people in intermediate class were smaller, details available on request.) In the baseline model (Figure 5a) the coefficient for the whole sample is 0.24 (95% CI 0.22 to 0.26).The working class (relative to salariat) coefficient for Nordic countries is above the European average whilst that for Southern European countries is below. There is not a consistent pattern for the remaining welfare states. Adjusting for financial strain attenuates class differences across the populations (Coefficient 0.13 95% CI 0.11 to 0.15). However, the degree of attenuation varies by country. The smallest attenuation occurs for Nordic countries (see Figure 5b) and largest in Southern European countries. Adjusting for labour force status had slightly smaller attenuating effect than financial strain reducing the mean European coefficient to 0.15 (95% ci 0.14 to 0.17) adjusting for labour forces status reduced the between country differences, with greater attenuation occurring for Nordic rather than Southern European countries (see Figure 5c). After adjusting for both labour force status and financial strain the coefficient for the whole sample was considerably reduced to 0.09 (95% CI 0.07 to 0.11). The consequences of adjusting for both labour force status and financial strain varied across countries. For Southern European countries the association between class and health was almost eliminated (see Figure 5d). However, the coefficients for all Nordic countries remained above the European average and were amongst the largest in the sample.

**Figure 6:**
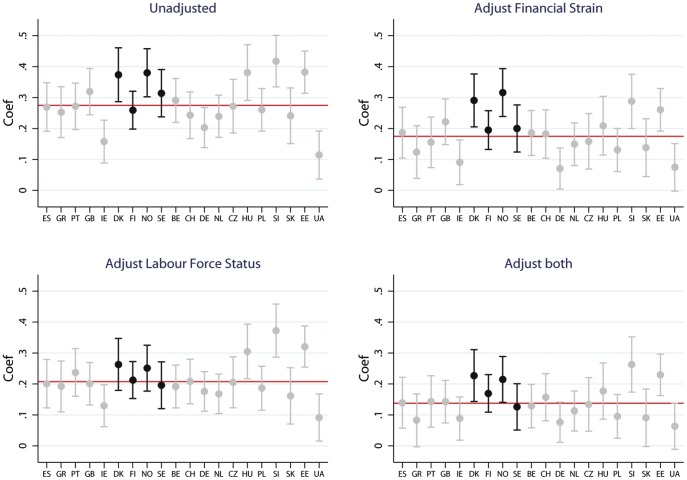
Self-rated health before and after adjustment, women. Figure 6 shows the predicted coefficients for self-rated health from marginal models for working class (relative to Salariat) for women for the entire sample (red line) and for 20 countries in the European Social Survey. Models include the base model, adjusting for financial strain, adjusting labour force status and adjusting for both labour force status and financial strain.

For working class women relative to salariat the average regression coefficient for the entire sample was 0.27 (95% CI 0.26 to 0.29). Coefficients for countries in the Southern European and Bismarkian regimes are closer to the European mean (see Figure 6a.) The Nordic countries divide into two groups. Denmark and Norway have large class differences whilst Finland and Sweden are close to the average for the sample. There is no consistent pattern for class differences in health for the other welfare regimes. Adjusting for financial strain reduced the working class coefficient for all countries to 0.17 (95% 0.16 to 0.19), with greater reductions for Southern and Easter Europe than for Nordic countries see Figure 6b. In contrast adjusting for Labour force status reduced the European coefficient to 0.21 (95% CI 0.20 to 0.22) with most countries except those in Eastern and Former Soviet regimes moving towards that average (see Figure 6c). Adjusting for both financial strain and labour force status lead to a reduction of the European mean class coefficient for women to 0.14 (95% CI 0.12 to 0.15), however, the country variations in class inequalities in health remained similar to the base model which did not adjust for labour force status and financial strain (see Figure 6d).

**Figure 7:**
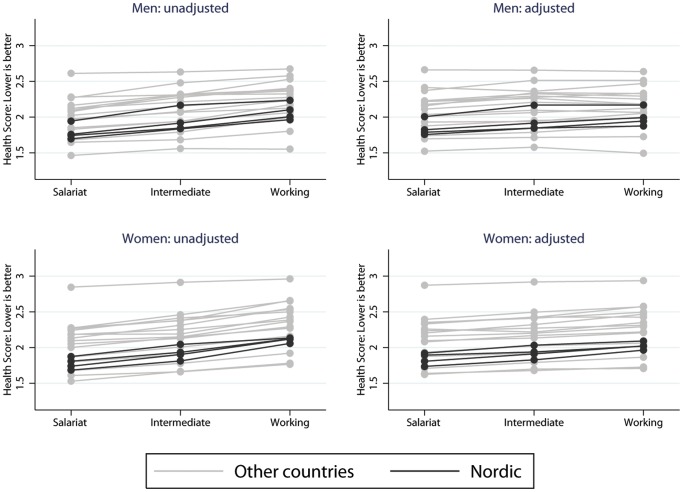
Health by class and country before and after adjustment. Figure 7 shows absolute predicted health scores produced by marginal standardisation from linear regression models by social class and country for men and women before and after adjusting for financial strain and labour force status.

When evaluating absolute levels of health men and women in Nordic countries had relatively good self-reported health, but by no means the best (see Figure 7). The countries with the best (Greece) and worst overall health (Ukraine) both had small inequalities. The predicted self-rated health scores by country and gender both before and after adjusting for financial strain and labour force status are in Table S1 (men) and Table S2 (women).

## Discussion

### Main findings

Our results show that class-based inequalities in health, financial strain and labour force status exist in countries with different welfare states. Across Europe over half of the class inequalities are explained by financial strain and labour force status. However, the proportion explained varies across the countries. It appears that for some countries, and particularly men in Southern Europe, that a very high proportion of class inequalities in health are attenuated by inequalities in financial strain and a lesser extent labour force status. However, this is not the case for Nordic countries where relatively large class based health inequalities remain after adjustment and if anything Nordic countries relative position worsened slightly after controlling for financial strain. Thus our results provide little support for the concept that relatively large class-based health inequalities in Nordic countries persist due to financial security and labour force status [2].

Whilst people living in Nordic countries did not have the best average health overall, all social classes had relatively good health and working class people in Nordic countries had health comparable if not better than salariat people living in Eastern Europe. Nordic countries have not eliminated class inequalities in financial security and non-employment but they have high levels of financial security and employment for all classes which may have improved health for all but not eliminated inequalities. The lower attenuating effect of financial strain in Nordic countries may partly be because the social gradient in financial strain for Nordic countries is of a somewhat different nature from other countries particularly the poorer Eastern European ones. The starkest contrast is with Ukraine which had the smallest health inequalities and the worst overall health where the majority of people in all classes were finding things difficult. In between are countries where people are more equally distributed throughout the financial strain categories, and it is for these countries that the highest proportion of working class disadvantage is explained by financial difficulty.

### Comparisons with other studies

Our results for Southern European countries are similar to those of Eikemo et al [20] who found small class based- inequalities in self-rated health for Southern Europe were explained by education and income. However, by investigating countries separately we go beyond Eikemo's et al analyses. Eikemo et al pooled 23 countries into 4 groups Eastern, Southern, Central and Northern. In particular, Eikemo's study included countries using either the Nordic or the Anglo-Saxon Welfare model within the same” Northern” regime, and found moderate attenuation of health inequalities for that regime. In contrast, we find that the degree of attenuation in class-based health inequalities varied across these welfare states with it tending to be small in the Nordic countries, but considerably greater in the United Kingdom.

Similar to our study, Aladbe et al [19] investigated if occupational class differences in health were explained by a variety of measures including financial problems which explained over a quarter of class differences in health, material deprivation which explained over half, and economic activity which lead to only lead to a small attenuation of class differences, 11% for men and 8% for women. However, Aladbe et al's study differed from ours in that their study pooled 28 countries from the European Quality of life Survey into a single sample. Given that economic activity classifications vary across countries [29], [34] by conducting analyses separately for each country our study may be able to more accurately assess the ability of economic activity measures to attenuate the relationship between social class and health.

### Interpretation and implications

In addition, to building on the existing literature which shows that health inequalities vary across Europe [8], [35]. We also find evidence to suggest that the mechanisms linking class and health vary across countries. It would appear that for Southern European countries that class differences in health are very small after adjusting for financial security and labour force status. This implicates mechanisms relating to income, welfare and the labour market as causes of health inequalities in Southern European countries. In stark contrast, for Nordic countries a substantial proportion of class inequalities in health remain unexplained after adjusting for financial strain and labour force status. Whilst Nordic welfare states have been good at promoting health generally our results would suggest that in Nordic countries have additional health risks linked to class that do not exist in Southern European countries. It is beyond the scope of our data to explore what these additional health risks are and many have been discussed elsewhere [1], [2]. However, one possibility is that whilst the core of the welfare state remains in Nordic countries increased liberalisation and flexibility of the labour market may have led to forms of precarious employment [12] which are not adequately captured by a cross-sectional labour force measures. Alternatively factors which are weakly socially patterned in Southern European countries, for example diet [10], may be strong candidates to explain inequalities in Nordic countries.

### Strengths and limitations

Our study has many strengths. It has a large sample size and uses measures of financial strain and class that are consistent across the 20 European countries enabling the investigation of countries with very different social policies. However, country variations in people's willingness to respond to surveys, has to be acknowledged. There are also country variations in the way in which people respond to questionnaires when rating their health [36], [37]. Whilst self-rated health has consistently been shown to be associated with morbidity and mortality [38], the same levels of health may not be comparable across countries [39]. Contextual factors will also be varying across time. This is a particular concern in relation to welfare states which are not static and will vary by the government of the day with some aspects being strengthened over time and others weakened [13]. By adjusting for survey rounds, which had a very small association with health of limited impact, we partially accounted for changes in contemporary contextual effects. However, without extensive longitudinal individual level data we are unable to investigate how age and period effects interact to create cohorts which may have very different experiences across the life course.

One of the limitations of this study is the ability to infer causality. Whilst financial strain and labour force status are plausible mechanisms for the relationship between class and health, these measures could also be indicators for other pathways linking class and health. For example financial strain may be an indicator of status which itself has been associated with health [40]. Inferring causality is also limited by the cross-sectional nature of our data. In particular we are unable to determine the extent to which class and labour force status are either a cause and/or a consequence of health. However, selection effects are likely to be limited in their ability to explain class inequalities as longitudinal research has shown that social mobility only explains a very small proportion of health inequalities [41]. Financial strain has many research advantages over income, it is easy to record, explain and simple to interpret [23] and has been associated with health measures notably depression [42], [43]. However, there may be some concern that the financial strain and subjective health measure are tapping into the same latent propensity to respond negatively to questionnaires. This is clearly not the case for all countries; people in Greece had both the best overall health, and also high levels of income strain.

Our theoretical framework has focused on social class – based on occupation - and how the health disadvantage of those in lower social classes, compared to higher, may be a consequence of their greater risk of labour market and financial disadvantage. There are alternate ways of conceptualising and operationalising socio economic position [44] and measuring inequalities [45]–[48]. Thus our study relates to explaining differences between social – occupation based – classes and differing mechanisms may apply for other concepts of social inequalities.

### Conclusion

Whilst financial security and labour force status play important roles in explaining class based health inequalities in many countries and in particular those of Southern and Eastern Europe, adjusting for financial security and labour force status leads to only modest reductions in health inequalities in Nordic countries. To understand the persistence of these inequalities we may need to look to other causes.

## Supporting Information

Table S1 **Health by class and country before and after adjustment, men.** Table S1 shows average health score by class and country before and after adjusting for financial strain and labour force status (men).
(DOCX)

Table S2 **Health by class and country before and after adjustment, women.** Table S2 shows average health score by class and country before and after adjusting for financial strain and labour force status (women).
(DOCX)
