# Supplementary material for: To What Extent Do Financial Strain and Labour Force Status Explain Social Class Inequalities in Self-Rated Health? Analysis of 20 Countries in the European Social Survey
Source: PLoS One. 2014 Oct 14;9(10):e110362. doi: 10.1371/journal.pone.0110362 (PMC4196960; doi:10.1371/journal.pone.0110362)
Supplement: Table S2 — Health by class and country before and after adjustment, women. Table S2 shows average health score by class and country before and after adjusting for financial strain and labour force status (women). (DOCX) [file pone.0110362.s002.docx]

Table S2: Health by class and country before and after adjustment, women

|  | Unadjusted^1^ | | | | | | | | | | |  | Adjusting Financial strain and labour force status^1^ | | | | | | | | | | |
| --- | --- | --- | --- | --- | --- | --- | --- | --- | --- | --- | --- | --- | --- | --- | --- | --- | --- | --- | --- | --- | --- | --- | --- |
|  | Salariat | | |  | Intermediate | | |  | Working | | |  | Salariat | | |  | Intermediate | | |  | Working | | |
|  | Av | 95% CI | |  | Av | 95% CI | |  | Av | 95% CI | |  | Av | 95% CI | |  | Av | 95% CI | |  | av | 95% CI | |
|  |  |  |  |  |  |  |  |  |  |  |  |  |  |  |  |  |  |  |  |  |  |  |  |
| Spain | 2.00 | 1.94 | 2.06 |  | 2.15 | 2.08 | 2.21 |  | 2.27 | 2.22 | 2.32 |  | 2.08 | 2.01 | 2.15 |  | 2.17 | 2.11 | 2.23 |  | 2.22 | 2.17 | 2.26 |
| Greece | 1.53 | 1.47 | 1.59 |  | 1.67 | 1.61 | 1.72 |  | 1.78 | 1.73 | 1.84 |  | 1.63 | 1.56 | 1.69 |  | 1.70 | 1.65 | 1.75 |  | 1.71 | 1.66 | 1.76 |
| Portugal | 2.26 | 2.20 | 2.33 |  | 2.38 | 2.32 | 2.44 |  | 2.54 | 2.49 | 2.58 |  | 2.35 | 2.28 | 2.42 |  | 2.41 | 2.35 | 2.48 |  | 2.50 | 2.45 | 2.54 |
| UK | 1.80 | 1.75 | 1.85 |  | 1.90 | 1.85 | 1.96 |  | 2.12 | 2.06 | 2.18 |  | 1.88 | 1.83 | 1.92 |  | 1.92 | 1.87 | 1.98 |  | 2.02 | 1.97 | 2.07 |
| Ireland | 1.61 | 1.56 | 1.66 |  | 1.66 | 1.61 | 1.72 |  | 1.77 | 1.72 | 1.82 |  | 1.64 | 1.59 | 1.69 |  | 1.68 | 1.62 | 1.73 |  | 1.73 | 1.68 | 1.77 |
| Denmark | 1.68 | 1.64 | 1.73 |  | 1.81 | 1.75 | 1.88 |  | 2.06 | 1.99 | 2.13 |  | 1.74 | 1.69 | 1.78 |  | 1.83 | 1.77 | 1.89 |  | 1.96 | 1.90 | 2.03 |
| Finland | 1.87 | 1.83 | 1.92 |  | 2.04 | 1.99 | 2.10 |  | 2.13 | 2.09 | 2.18 |  | 1.92 | 1.88 | 1.96 |  | 2.03 | 1.98 | 2.09 |  | 2.09 | 2.05 | 2.13 |
| Norway | 1.74 | 1.69 | 1.79 |  | 1.90 | 1.84 | 1.96 |  | 2.12 | 2.06 | 2.18 |  | 1.81 | 1.76 | 1.85 |  | 1.91 | 1.86 | 1.97 |  | 2.02 | 1.97 | 2.08 |
| Sweden | 1.81 | 1.76 | 1.86 |  | 1.93 | 1.87 | 2.00 |  | 2.12 | 2.06 | 2.18 |  | 1.89 | 1.85 | 1.94 |  | 1.94 | 1.88 | 2.00 |  | 2.02 | 1.97 | 2.08 |
| Belgium | 1.87 | 1.82 | 1.91 |  | 2.01 | 1.95 | 2.06 |  | 2.16 | 2.10 | 2.22 |  | 1.93 | 1.89 | 1.98 |  | 2.03 | 1.97 | 2.08 |  | 2.06 | 2.01 | 2.11 |
| Switzerland | 1.68 | 1.64 | 1.72 |  | 1.78 | 1.73 | 1.83 |  | 1.92 | 1.86 | 1.99 |  | 1.71 | 1.67 | 1.75 |  | 1.79 | 1.74 | 1.84 |  | 1.87 | 1.80 | 1.93 |
| Germany | 2.18 | 2.14 | 2.23 |  | 2.25 | 2.21 | 2.30 |  | 2.39 | 2.34 | 2.43 |  | 2.24 | 2.19 | 2.28 |  | 2.27 | 2.23 | 2.32 |  | 2.31 | 2.27 | 2.36 |
| Netherlands | 2.05 | 2.02 | 2.09 |  | 2.13 | 2.08 | 2.18 |  | 2.29 | 2.23 | 2.35 |  | 2.10 | 2.06 | 2.13 |  | 2.13 | 2.09 | 2.18 |  | 2.21 | 2.16 | 2.26 |
| Czech Repub | 2.10 | 2.03 | 2.16 |  | 2.15 | 2.09 | 2.21 |  | 2.37 | 2.31 | 2.43 |  | 2.16 | 2.09 | 2.23 |  | 2.20 | 2.14 | 2.26 |  | 2.29 | 2.24 | 2.35 |
| Hungary | 2.27 | 2.20 | 2.35 |  | 2.46 | 2.38 | 2.54 |  | 2.65 | 2.60 | 2.71 |  | 2.39 | 2.32 | 2.47 |  | 2.50 | 2.43 | 2.57 |  | 2.57 | 2.52 | 2.62 |
| Poland | 2.24 | 2.19 | 2.29 |  | 2.41 | 2.36 | 2.47 |  | 2.50 | 2.45 | 2.55 |  | 2.33 | 2.28 | 2.38 |  | 2.43 | 2.38 | 2.48 |  | 2.43 | 2.38 | 2.47 |
| Slovenia | 2.13 | 2.08 | 2.19 |  | 2.31 | 2.24 | 2.38 |  | 2.55 | 2.49 | 2.61 |  | 2.20 | 2.15 | 2.26 |  | 2.32 | 2.25 | 2.39 |  | 2.47 | 2.40 | 2.53 |
| Slovakia | 2.19 | 2.12 | 2.26 |  | 2.19 | 2.12 | 2.27 |  | 2.43 | 2.37 | 2.48 |  | 2.26 | 2.19 | 2.33 |  | 2.22 | 2.15 | 2.29 |  | 2.35 | 2.30 | 2.40 |
| Estonia | 2.28 | 2.23 | 2.33 |  | 2.38 | 2.30 | 2.45 |  | 2.66 | 2.61 | 2.71 |  | 2.35 | 2.30 | 2.39 |  | 2.42 | 2.35 | 2.49 |  | 2.58 | 2.53 | 2.62 |
| Ukraine | 2.85 | 2.79 | 2.90 |  | 2.91 | 2.82 | 3.01 |  | 2.96 | 2.91 | 3.01 |  | 2.87 | 2.82 | 2.93 |  | 2.92 | 2.83 | 3.01 |  | 2.94 | 2.89 | 2.99 |

1 All models includes adjusted for age and survey round.
